# Study of the antagonistic relationship between gene expression biofilm of *Aspergillus niger* and *Staphylococcus aureus* that cause otomycosis

**DOI:** 10.22034/cmm.2024.345248.1586

**Published:** 2024-12-31

**Authors:** Mouna Akee Hamed Al-Oebady

**Affiliations:** 1 Biology Department, College of Science, Al-Muthanna University, Samawah, Iraq

**Keywords:** Antagonistic, *Aspergillus niger*, eglA, eglB, eglC, eng1, exo, *Staphylococcus aureus*, xynB

## Abstract

**Background and Purpose:**

Various species of microorganisms interact in a variety of ecological niches and can lead to infection. A biofilm of one or more species may form during the infectious process. Otomycosis can be brought on by etiologic agents, such as *Staphylococcus aureus* and *Aspergillus niger*. This study aimed to survey the antagonistic relationship between the gene expression biofilms of *A. niger* and *S. aureus* in the context of otomycosis-related biofilm formation.

**Materials and Methods:**

This study examined single-species biofilms of *A. niger* and *S. aureus*, as well as mixed-species biofilms of *A. niger*-*S. aureus*, over 24 and 48 h. Expression of *A. niger* biofilm-related genes (*eng1*, *xynB*, *exo*, *eglA*, *eglB*, and *eglC*) was analyzed using real-time polymerase chain reaction (PCR). Impact of *S. aureus* on the gene expression of *A. niger* was evaluated and compared to the gene expression of *A. niger* alone, which served as the control.

**Results:**

Biofilm formation assays showed that *A. niger* biofilm formation was significantly inhibited when co-cultured with *S. aureus*, with optical density values dropping from 0.56 (alone) to 0.15 at 24 h and 0.05 at 48 h. Real-time PCR analysis revealed that the expression of *A. niger* biofilm-related genes, namely *eng1*, *xynB*, *exo*, *eglA*, *eglB*, and *eglC*, increased significantly in single-species biofilms, reaching 2.5, 3, 1.5, 3.5, 2, and 1.7, respectively, at 24 h and 3.5, 4, 2, 4.2, 3, and 2, respectively at 48 h. However, in co-culture with *S. aureus*, their gene expression was markedly reduced to 0.8, 0.5, 0.4, 0.9, 0.6, 0.5, respectively, at 24 h and 0.5, 1, 0.2, 0.8, 0.6. , and 0.3, respectively, at 48 h, demonstrating a strong inhibitory effect of *S. aureus* on *A. niger* biofilm formation and gene expression.

**Conclusion:**

This study described the antagonistic relationship between *S. aureus* and *A. niger* on the gene expression biofilm that causes otomycosis, as well as the antibiosis relationship between the two during in vitro biofilm formation. These findings provide new insights into the complex interactions between these microorganisms during infection and may have implications for understanding and managing otomycosis.

## Introduction

otomycosis is a fungal infection that can range from acute to chronic. It is commonly mistaken secondary infection of the outer ear, which for a includes the auricle and external auditory canal [ 1 ]. In certain instances, this illness also affects the tympanic membrane due to disease extension. However, the agents that cause otomycosis rarely infect the middle ear. Most typical symptoms of this disease include gradual hearing loss accompanied by ear fullness, itching, pain, redness, swelling, and discharge [ 2 ]. While the structure of the ear canal serves as a d home for fungi, otomycosis is predisposed pathway anby tropical weather, injuries, bacterial otitis, ear aids abnormalities, and hearing [ 3 ].

Biofilms are microbial communities of cells, which adhere to surfaces and are embedded in an extracellular polymeric matrix that the organism produces on its own. This matrix creates a suitable protective environment for development and endurance in harsh environments [ 4 ]. Planktonic and biofilm cells display a unique biological characteristic that sets them apart from their natural resistance to antimicrobials and immune assault [ 5 ].

Support structure of the biofilm is created by a planktonic species that sticks to a surface and starts to form structural scaffolds. This process is called surface colonization by one of the constituent species. Moreover, co-aggregation is the term for this sequential adhesion process [ 6 ]. Consortia of the two interacting microorganisms form the mixed fungal-bacterial biofilm. Contact and adhesion are the fundamental processes for the formation of polymicrobial biofilms in fungal-bacterial interactions [ 7 ].

Three stages of attachment, maturation, and dispersion are normally associated with the formation of biofilms, which are highly structured microbial communities [ 8 ]. Nevertheless, due to the distinct morphology and purpose of their spores and hyphae, filamentous fungi produce biofilms in a different way than bacteria and yeast [ 9 ]. Development stage of filamentous fungi in biofilms includes, in particular, spore attachment, germination, hyphal elongation, colonization, extracellular matrix synthesis, maturation, and diffusion of biofilms [ 10 ]. Formation of biofilms by filamentous fungi, which involves complex interactions between biological and physical processes, is often dependent on the ability of spores to attach. Second, during the intermediate stage of biofilm formation, hyphae cross-linking is crucial for the synthesis of extracellular matrix (ECM). Fungal filamentous biofilms are produced as a result, distinct from those of bacteria and yeast [ 11 ].

 Involvement of biofilms in co-infections has been connected to virulence factors in the medical domain, such as adhesion mechanisms and the synthesis and secretion of proteins, toxins, and enzymes [ 12 ]. There has been a surge in research on polymicrobial biofilms. Consequently, it is also believed that the development of biofilms within the host is a determinant virulence factor for pathogenesis [ 13 ]. *In vitro* mixed biofilm formation between *Staphylococcus aureus* and *Aspergillus niger* has been studied with an emphasis on their ecological interactions. This study aimed to describe some of the mechanisms behind this underexamined interaction, which appears to be an antibiosis effect of *S. aureus* on *A. niger*, according to analysis of mixed biofilms of these microbes. 

## Materials and Methods

### Samples collection

The study population consisted of patients with otomycosis who referred to Al Hussein Teaching Hospital in Samawah City, Iraq. For the purposes of the study, *A. niger* and *S. aureus* were isolated from the patients. *A. niger* isolate was cultured in potato dextrose agar (PDA) medium at 37 °C for five days. Brain Heart Infusion (BHI) agar plates were seeded with the *S. aureus* isolate by cross-streaking at 37 °C, and the plates were then incubated for 24 h.

### Microbiological and molecular identification

Clinical isolates of *S. aureus* and *A. niger* were identified using biochemical profiles, colony and microscopic morphology, and microbiological techniques [ 14 , 15 ]. Genomic DNA for both isolates was obtained using the method [ 16 ] for molecular identification.

Through the usage of universal primers, namely fD1 and rD1, the 16S rDNA gene sequence was amplified in order to identify *S. aureus*, following the guidelines provided by Weisburg et al. [ 17 ]. Amplification procedure outlined by Gardes et al. [ 18 ] was followed in order to identify *A. niger* using the fragment ITS1-5.8S rDNA-ITS2. The software known as Basic Local Alignment Search Tool (BLAST) was also used to compare the nucleotide sequences.

### Formation and quantification of biofilm

Single and mixed biofilms of *A. niger* and *S. aureus* were grown on 96-well flat bottom polystyrene plates. Conidia from the aerial static culture were taken for the *A. niger* biofilm and suspended in RPMI medium with 2% glucose added, in accordance with the procedure described by Mowat et al. [ 19 ]. The ideal biofilm for *S. aureus* and *A. niger* was determined to be 1 × 105 conidia/mL and 1 × 108 bacteria/mL, respectively, based on initial experiments. For the purpose of forming biofilms, 100 μL of conidial and/or bacterial suspension in RPMI supplemented with 2% glucose was introduced into every well. The non-adherent cells were eliminated by discarding the supernatant following 4 h of incubation at 37 °C (adherence stage), and 200 μL of fresh RPMI medium was added. Plates were then left to incubate for 0, 4, 8, 16, and 24 h. Upon removal of the medium, the wells were cleaned using 200 μL of phosphate buffered saline. Usage of crystal violet at 0.005%, biofilm biomass was measured indirectly using the technique described by Peeters et al. [ 20 , 21 , 22 ]. After the removal of the excess dye with distilled water, the area was allowed to air dry. Afterward, 200 μL of acetic acid was added at 33% (v/v) for 15 min to dissolve the dye attached to the biofilm. The resulting solution was then moved to a 96-well microtiter plate, cleaned, and measured at 595 nm using a microplate reader. Amount of biomass in the biofilm is directly correlated with the optical density (OD) values. Three separate runs of the experiment were conducted using 10 different biofilms for the same model.

Following the previous instructions, the following changes were made to the mixed biofilm: the fungus and the bacteria were inoculated separately and allowed to incubate for 4 h. The microorganism that had not been involved in the interaction between the fungus and the bacteria was then added, and it was incubated for 24 h at 37°C.

### RNA isolation and reverse transcription-polymerase chain reaction

The total RNA was isolated from the samples using the RNeasy Plant Mini Kit (QIAGEN, Tokyo, Japan) in compliance with the instructions of the manufacturer. On total RNA, reverse transcription was carried out using the Omniscript RT kit (QIAGEN, Tokyo, Japan). Each reaction tube received the following additions: 1 µL of reverse transcriptase, 1 µL of RNase inhibitor (10U/mL), 2 µL of 10X RT buffer, 2 µL of dNTP mix (5 mM each), and 12 µL of total RNA (2 µg). The reaction was run at 37 °C for 1 h. The reverse transcription-polymerase chain reaction (RT-PCR) was performed using synthesized cDNA. The primer combinations shown in Table 1 were obtained from the *A. niger* data bank of the National Center of Biotechnology Information (www.ncbi.nlm.nih.gov). The RT-PCR was used to test for the exponential phase after cycle optimization. Each tube held the following: 1.5 µL of cDNA sample, 10.12 µL of ultra-pure water, 0.08 µL of Gen Taq polymerase (5U/µL; WaKo), 1.2 µL of dNTP mix (5 mM each), and 0.3 µL of forward primer (10 µM), and 0.3 µL of reverse primer (10 µM). The reaction process was as follows: step 1 (30 s) 95 °C, step 2 (30 s) 55 °C, step 3 (30 s) 72 °C, cycle 1 (1x) 95 °C (4 min), cycle 2 (40x), and cycle 4 (1x) 4 °C until use.

**Table 1:** Polymerase chain reaction primers for real-time reverse transcriptase

Genes	Forward	Reverse
*Eng1*	5’ cgacttggtcagtttgatacc 3’	5’ atacccgtgtaagcagttcc 3’
*Xynb*	5 agcggatcatgggaaaccga 3’	5’ gtgtaatctatgaatgcctatagcgggtaa 3’
*Exo*	5’ tgtgctctcgttgccctcttg 3’	5’ agtgcattggcgccttcctc 3’
*EglA*	5’ tccccgtgtcacttgctatg 3’	5’ cagttcatagtcgccgctaga 3’
*EglB*	5’ atctcaaccaagcagccatt 3’	5’ ccaggatatccagcataccc 3’
*Eglc*	5’ tggtgttaccggtctcttcaaaaccga 3’	5’ gctataccagggatagacttacactgcgaa 3’

### Statistical Analysis

Different means of biofilm biomass (absorbance) were compared using the t-test. Moreover, data analysis was performed in GraphPad software.

## Results

### Molecular and microbiological identification

Clinical isolate of *A. niger* developed the morphologic features of this species in five days at 37 °C on PDA medium [ 23 ]. After molecular analysis, the ITS nucleotide sequence (600 bp) of *A. niger* showed 100% homology with sequences reported for this fungus in the GenBank using the BLAST. Following a 24-hour growth period at 37 °C on BHI agar, the clinical *S. aureus* isolate exhibited species characteristics [ 24 ]. The 16S rDNA gene sequence (1500 bp) of the isolated *S. aureus* showed 99% homology with the sequences reported for *S. aureus*.

### Biofilm formation

**Figure 1:**
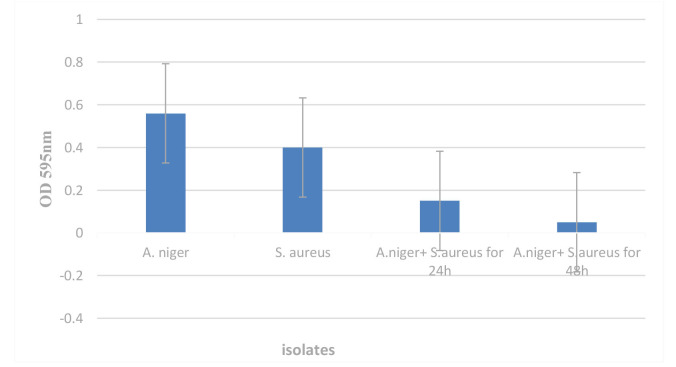
Biofilm formation of *Aspergillus niger* in co-culture with *Staphylococcus aureus* for 24 h and 48 h.

Biofilm for *A. niger* was incubated alone and when it was incubated along with *S. aureus*, they were compared during 24 h and 48 h. When *A. niger* was co-cultured with *S. aureus* for 48 h, its biofilm formation ability was lower than what it was at 24 h. The (OD) of *A. niger* culture alone was OD595=0.56. While, OD595=0.4 for *S. aureus* and co-cultured *A. niger* with *S. aureus* were 0.15 and 0.05 for 24 h and 48 h, respectively (Figure 1). 

### Transcriptase real-time polymerase chain reaction

Reverse transcription real-time PCR was used to measure the relative expression of the implicated genes (*eng1*, *xynB*, *exo*, *eglA*, *eglB*, and *eglC*) in *A. niger* over the course of the biofilm experiment for 24 h.

**Figure 2:**
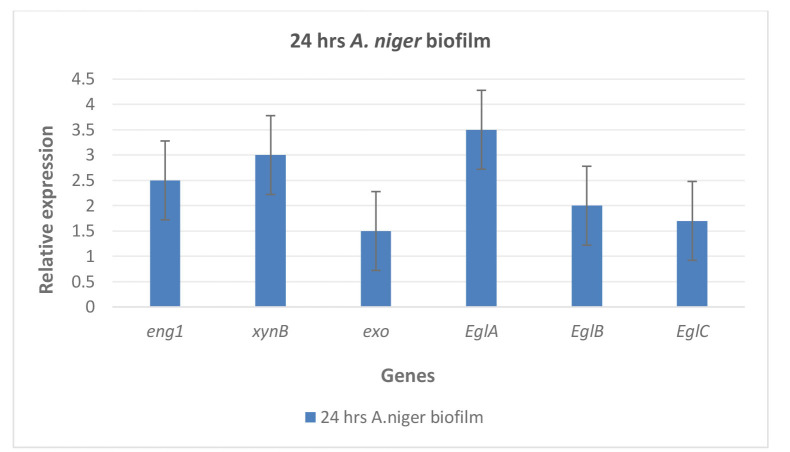
Relative expression of the implicated genes (*eng1*, *xynB*, *exo*, *eglA*, *eglB*, and *eglC* ) in *A. niger* during the biofilm experiment which was considered the control for 24 h using reverse transcription real-time polymerase chain reaction.

Expression of the biofilm genes developed by *A. niger* was evaluated by quantitative reverse transcriptase PCR in this study. Biofilm genes expression of *A. niger*, *eng1*, *xynB*, *exo*, *eglA*, *eglB*, and *eglC* were 2.5, 3, 1.5, 3.5, 2, and 1.7, respectively. It underwent a dramatic increase in expression over time and was considered a measure of control when compared with the genes encoding the biofilm with *S. aureus* (Figure 2).

The implicated genes (*eng1*, *xynB*, *exo*, *eglA*, *eglB*, and *eglC*) were measured for relative expression using real-time RT-PCR in *A. niger* during the course of the 48-h biofilm experiment.

**Figure 3:**
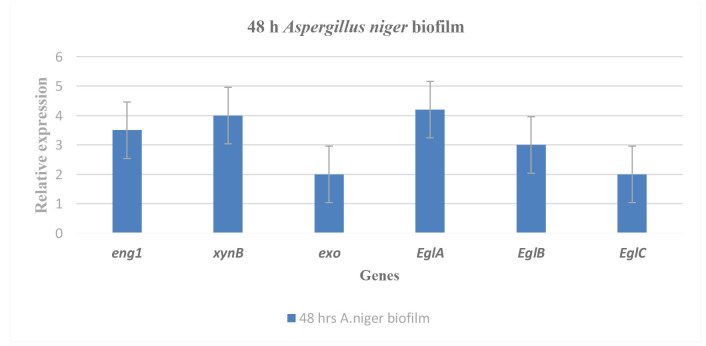
Reverse transcription real-time polymerase chain reaction was used to measure the relative expression of the implicated genes (*eng1*, *xynB*, *exo*, *eglA*, *eglB*, and *eglC*) in *Aspergillus niger* during the biofilm experiment and it was considered as the control for 48 h.

In this study, quantitative reverse transcriptase PCR was used to assess the expression of the biofilm genes produced by *A. niger*. Compared to the genes encoding the biofilm with *S. aureus*, the expression levels of the biofilm genes of *A. niger*, namely *eng1*, *xynB*, *exo*, *eglA*, *eglB*, and *eglC* were 3.5, 4, 2, 4.2, 3, and 2, respectively. These biofilm genes showed a significant increase in expression over time (Figure 3).

**Figure 4:**
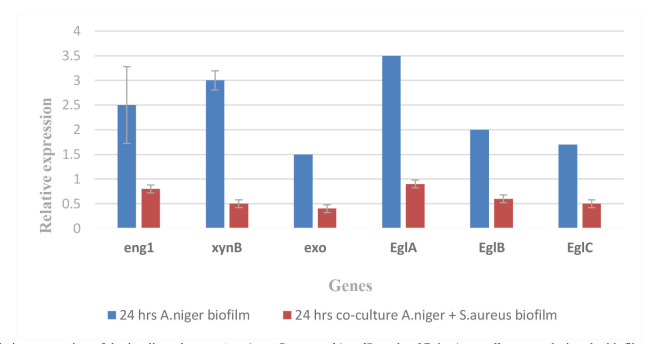
Relative expression of the implicated genes (*eng1*, *xynB*, *exo*, *eglA*, *eglB*, and *eglC*) in *Aspergillus niger* during the biofilm experiment and co-culture of *A. niger* with *S. aureus* biofilm using reverse transcription real-time polymerase chain reaction for 24 h (t=5.3402, df=10, standard error of difference=0.328, P<0.05).

According to the results of real-time PCR using the specific primers (*eng1*, *xynB*, *exo*, *eglA*, *eglB*, and *eglC*), *A. niger* co-culture with *S. aureus* biofilm had less biofilm formation during 24 h. Figure 4 illustrates the *eng1* (0.8), *xynB* (0.5), *exo* (0.4), *eglA* (0.9), *eglB* (0.6), and *eglC* (0.5) genes expressions in *A. niger* co-culture with *S. aureus* biofilm. This indicates the inhibitory role of *S. aureus* on the *A. niger* biofilm within 24 h, compared to the control gene expression.

**Figure 5:**
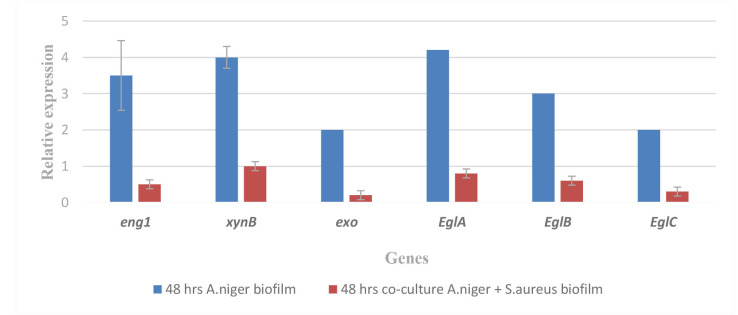
Relative expression of the implicated genes (*eng1*, *xynB*, *exo*, *eglA*, *eglB*, and *eglC*) in *Aspergillus niger* during the biofilm experiment and co-culture of *A. niger* with *S. aureus* biofilm using reverse transcription real-time PCR for 48 h. (t=6.2080, df=10, standard error of difference=0.411, P<0.05).

Results from a real-time PCR using the particular primers (*eng1*, *xynB*, *exo*, *eglA*, *eglB*, and *eglC*) showed that *A. niger* co-culture with *S. aureus* biofilm had less biofilm formation during 48 h. Figure 5 illustrates the *eng1* (0.5), *xynB* (1), *exo* (0.2), *eglA* (0.8), *eglB* (0.6), and eglC (0.3) genes expressions in *A. niger* co-culture with *S. aureus* biofilm. These results are conclusive evidence that *S. aureus* is a highly efficient inhibitor of *A. niger* biofilm. Based on these results, *S. aureus* is effective on the genes responsible for the formation of the biofilm of *A. niger* (Figure 5).

## Discussion

Bacteria and fungi are among the many species that comprise microbial consortia. On rare occasions, they may induce infections that proliferate by adhering to host cells, aggregating, colonizing, and producing an ECM composed of exopolymers before the eventual formation of biofilms [ 25 , 26 ]. Polymicrobial otomycosis, which is brought on by an infection with fungus bacteria, serves as an illustrative example [ 27 ].

To the best of our knowledge, the present study was the first to report the antibiosis effect of *S. aureus* on *A. niger* on mixed biofilm assessment by gene expression. *In vitro* biofilm formation between clinical isolates of *S. aureus* and *A. niger* from patients with microbial otomycosis revealed the antagonistic nature of their interaction. Regardless of the stage of biofilm formation, damage to fungal structures, and gene expression of *A. niger* biofilm, *S. aureus* consistently inhibited the mixed biofilm formed by *A. niger*-*S. aureus*.

Single biofilms were compared with those found in the mixed biofilm to evaluate the changes brought about by *S. aureus* to *A. niger*. Overall, Figure 1 shows that the amount of *A. niger* biofilm was lower than those of *S. aureus* and mixed biofilm. This effect may be due to the growth type, metabolic activity, and cell morphological variation, which are the characteristics that set bacterial biofilms apart from fungal ones [ 28 ].

Biofilm of *A. niger* is formed by asynchronous fungal growth, which allows conidia derived from mature hyphae to continue germination into new generation hyphae. Channels are specialized structures used for the removal of toxic metabolites and the transportation of nutrients and water. This process makes it possible for channels to form [ 29 ]. Moreover, extracellular polymeric substance secretion is a crucial characteristic of fungal biofilms.

Compact microcolonies releasing ECM were evident in the *S. aureus* biofilm. Additionally, it has been documented that the formation of those polymeric bridges enables bacterial attachment or linking via electrostatic and Van der Waals forces, among other ECM-surface interactions [ 30 ]. Bacterial antagonism on *A. niger* during 24 and 48 h was indicated by the RT-PCR, which revealed reduced germination of the fungus genes in the mixed biofilm (Figures 4-5). Nevertheless, the bacterial or fungal origin of these pleomorphic cells requires further investigation. 

Figure 1 shows the antibiosis of *S. aureus* on mixed biofilm of *A. niger*. Throughout 24 and 48 h, it was observed that *A. niger* reduced the formation of biofilms and inhibited fungal growth, even at low concentrations of *S. aureus*. 

In light of these findings, the authors of the present study suggest that a product of *S. aureus* may have contributed to the demise of *A. niger*. These bacterial components most likely have enzymatic activity since *S. aureus* uses enzymes to induce metabolic disequilibrium in Cryptococcus neoformans [ 31 ]. Nonetheless, the possibility of secretion of a variety of chemicals with distinct functions by *S. aureus* increases the likelihood that they are exotoxins. Mixed biofilm of *Aspergillus fumigatus* and *Pseudomonas aeroganosa* exhibited similar antagonistic effects, which led to the conclusion that extracellular molecules of bacterial origin, which were diffusible, were responsible for the effect [ 31 ].

Formation of mixed biofilm is dependent on the adhesion of the primary colonizer. Therefore, in order to assess the antagonistic effect of *S. aureus* on *A. niger* during the adhesion stage in the mixed biofilm, assays were performed in which the primary colonizer was alternated and the inoculum concentration, adhesion time, and gene expression were varied.

## Conclusion

In conclusion, to the best of our knowledge, this is the first study reporting that *S. aureus* has an antagonistic effect on the expression of the *A. niger* biofilm gene during fungal development. Conidiation, filamentation, and subsequent biofilm formation of *A. niger* are inhibited by *S. aureus* during the mixed biofilm formation process. By producing bacterial products and presumably through cell-to-cell contact, the bacterium severely restricts fungal growth. This incident may be connected to other published studies and clinical observations made by ear doctors in cases of infectious otomycosis, where they discovered that a mixed infection (fungus and bacteria) led to a better clinical evolution. Therefore, these results provide clinical data for research on treatments for otomycosis and may be used as therapeutic substitutes.

## References

[1] Younas AT, Al-Kataan MA, Al-Rejabooa MA ( 2021). Detection of some virulence factors of fungi caused Otomycosis isolated from some hospitals and clinics in Mosul/Iraq. Al-Qadisiyah Journal of Pure Science.

[2] Kamali Sarvestani H, Seifi A, Falahatinejad M, Mahmoudi S ( 2022). Black aspergilli as causes of otomycosis in the era of molecular diagnostics, a mini-review. J Mycol Med.

[3] Tasi´c-Otaševi´c S, Golubovi´c M, Ðeni´c S, Ignjatovi´c A, Stalevi´c M, Momˇcilovi´c S, Bojanovi´c M, Arsi´c-Arsenijevi´c V ( 2020). Species distribution patterns and epidemiological characteristics of otomycosis in Southeastern Serbia. J Mycol Med.

[4] Costerton J W, Stewart P S, Greenberg E P ( 1999). Bacterial biofilms: a common cause of persistent infections. Science.

[5] d’Enfert C, Janbon G ( 2016). Biofilm formation in Candida glabrata:what have we learnt from functional genomics approaches?. FEMS Yeast Res.

[6] Peters BM, Jabra-Rizk MA, O’May GA, Costerton JW, Shirtliff ME ( 2012). Polymicrobial interactions: impact on pathogenesis and human disease. Clin Microbiol Rev.

[7] Frey-Klett P, Burlinson P, Deveau A, Barret M, Tarkka M, Sarniguet A ( 2011). Bacterial-fungal interactions: hyphens between agricultural, clinical, environmental, and food microbiologists. Microbiol Mol Biol R.

[8] Reynolds TB, Fink GR ( 2001). Bakers’ yeast, a model for fungal biofilm formation. Science.

[9] Manfiolli AO, Dos Reis TF, de Assis LJ, de Castro PA, Silva LP, et al ( 2018). Mitogen activated protein kinases (MAPK) and protein phosphatases are involved in Aspergillus fumigatus adhesion and biofilm formation. Cell Surface.

[10] Mowat E, Butcher J, Lang S, Williams C, Ramage G ( 2007). Development of a simple model for studying the effects of antifungal agents on multicellular communities of Aspergillus fumigatus. J Med Microbiol.

[11] Kvasničková E, Paulíček V, Paldrychová M, Ježdík R, Maťátková O, Masák J ( 2016). Aspergillus fumigatus DBM 4057 biofilm formation is inhibited by chitosan, in contrast to baicalein and rhamnolipid. World J Microb Biotechnol.

[12] Yang L, Zheng C, Chen Y, Shi X, Ying Z, Ying H ( 2019). Nitric oxide increases biofilm formation in Saccharomyces cerevisiae by activating the transcriptional factor Mac1p and thereby regulating the transmembrane protein Ctr1. Biotechnol Biofuels.

[13] Liu L, Yu B, Sun W, Liang C, Ying H, Zhou S, et al ( 2020). Calcineurin signaling pathway influences Aspergillus niger biofilm formation by affecting hydrophobicity and cell wall integrity. Biotechnol Biofuels.

[14] Johnson EM, Borman AM, Pasqualotto A ( 2010). The importance of conventional methods: microscopy and culture. Aspergillosis: From Diagnosis to Prevention.

[15] De Cueto M, Pascual A, Pahissa A ( 2009). Microbiología y patogenia de las infecciones producidas por Staphylococcus aureus. Infecciones producidas por Staphylococcus aureus.

[16] Allers T, Lichten M ( 2000). A method for preparing genomic DNA that restrains branch migration of Holliday junctions. Nucleic Acid Res.

[17] Weisburg WG, Barns SM, Pelletier DA, Lane DJ ( 1991). 16S Ribosomal DNA amplification for phylogenetic study. J Bacteriol.

[18] Gardes M, Bruns TD ( 1993). ITS primers with enhanced specificity for basidiomycetes–application to the identification of mycorrhizae and rusts. Mol Ecol.

[19] Mowat E, Rajendran R, Williams C, McCulloch E, Jones B, Lang S, et al ( 2010). Pseudomonas aeruginosa and their small diffusible extracellular molecules inhibit Aspergillus fumigatus biofilm formation. FEMS Microbiol Lett.

[20] Christensen GD, Simpson WA, Younger JJ, Baddour LM, Barrett FF, Melton DM, et al ( 1985). Adherence of coagulase-negative staphylococci to plastic tissue culture plates: a quantitative model for the adherence of staphylococci to medical devices. J Clin Microbiol.

[21] Peeters E, Nelis HJ, Coenye T ( 2008). Comparison of multiple methods for quantification of microbial biofilms grown in microtiter plates. J Microbiol Methods.

[22] Ramírez-Granillo A ( 2013). Structural analysis of the in vitro biofilm formation by Staphylococcus aureus and Aspergillus fumigatus. Master thesis.

[23] Powell KA, Renwick A, Peberdy JF ( 2013 Jun 29). The genus Aspergillus: from taxonomy and genetics to industrial application.

[24] MAH Al-Oebady, AAA Dakl, HM Nahab ( 2019). influence of Staphylococcus aureus on the oral Candida albicans. Journal of Global Pharma Technology.

[25] Anggraini W, Purwanto DA, Kusumawati I, Isnaeni, Suryanto ( 2023). Influence of the Environment on Biofilm Formation Candida albicans of Vulvovaginal Candidiasis Isolate Patient. Pharmacognosy J.

[26] Masfufatun M, Purbowati R, Arum NA, et al ( 2022). An intestinal Candida albicans model for monomicrobial and polymicrobial biofilms and effects of hydrolases and the Bgl2 ligand. Vet World.

[27] te Biesebeke R, Levasseur A, Boussier A, Record E, Van den Hondel CA, Punt PJ ( 2010). Phylogeny of fungal hemoglobins and expression analysis of the Aspergillus oryzae flavohemoglobin gene fhbA during hyphal growth. Fungal Biol.

[28] Asih DW, Widodo ADW, Setiabudi RJ, et al ( 2023). Biofilm formation by the interaction of fungi (Candida tropicalis) with various bacteria. J Adv Biotechnol Exper Therapeut.

[29] Villena GK, Gutiérrez-Correa M ( 2007). Morphological patterns of Aspergillus niger biofilms and pellets related to lignocellulolytic enzyme productivities. Lett App Microbiol.

[30] Ahmed SH, Abdullah Z G, Sulaiman SS ( 2023). Biofilms formation and relationship to gene-producing biofilms in Staphylococcus aureus Isolated from clinical specimens. J Popul Ther Clin Pharmacol.

[31] Ikeda R, Saito F, Matsuo M, Kurokawa K, Sekimizu K, Yamaguchi M, et al ( 2007). Contribution of the mannan backbone of cryptococcal glucuronoxylomannan and a glycolytic enzyme of Staphylococcus aureus to contact-mediated killing of Cryptococcus neoformans. J Bacteriol.

